# Earlswood Asylum

**Published:** 1904-07-02

**Authors:** C. C. P. Hull, C. Holman, H. Howard

**Affiliations:** Chairman; 36 King William Street, E.C.; Member of Board of Management; 36 King William Street, E.C.; Secretary. 36 King William Street, E.C.


*To the Editor of* The Hospital.

### EARLSWOOD ASYLUM.

*To the Editor of* The Hospital.

Sir,—More than fifty years ago the need of an institution to provide for idiot and imbecile children was keenly felt, with the result that the asylum at Earlswood was founded. Since then the institution has been open to the children of all classes: the rich have been enabled to pay for that efficient care and proper tuition which only such an asylum can provide, and the poor have been relieved of a burden which weighs heavily on a hard-worked woman—the charge of a useless, perhaps noisy, uncleanly, or mischievous child. The incubus becomes even greater when the child is also an epileptic, when the scenes witnessed by the other children in the home are very demoralising. The founders of the asylum were the pioneers in this work, and a great want has been supplied. To curtail the numbers of the inmates—nearly 500—would be to inflict a great social, one could fairly say a national, injury.

Now, however, a crisis has occurred in the history of the asylum, owing to considerable structural defects which call for immediate attention. These, as in the case of other similar institutions, are the result of faulty work in the erection of the original buildings, in times when the supervision of local authorities was not as it is now. The asylum stands upon the Wealden clay. Practically no foundation was provided, and the walls were filled with rubbish; hence, owing to successive wet seasons, the whole building began to subside with the result that the walls cracked. Eminent architects were at once summoned, and they insisted that, for the safety of the inmates, the repairs should be immediately taken in hand, at an estimated cost of £30,000.

The funds available for these necessary operations are practically exhausted, so that, unless immediate substantial help is forthcoming, the progress of the work must be stopped. In this case the committee will have to do what they can to make the buildings secure for the approaching winter, and the cost of resuming the work next year will be greatly increased—for it must be done. Already a sum of £12,000 has been spent, and the labour bill is still £300 a week. No legal redress is possible after such a lapse of time.

The asylum had been placed in a position of comparative financial security, but now all available securities have been sold, and the institution is heavily in debt to the bankers. The public must at times experience difficulty in deciding which charitable institutions to select for their beneficence, but surely no eloquent voice is needed to plead for those who cannot plead for themselves. As the institution is open to all classes, and as no question is raised as to denomination, or even nationality, the committee have confidence in appealing to the public in general.

No one can deny that the immediate good done by the asylum in the past has been great; and it is difficult to estimate the potential good done to the community at large in fostering any germs of intelligence that may be found in the patients, with the result that many children who were worse than useless at home have been returned in the course of a few years to prove a comfort to the family, and even to become breadwinners.

Your faithful servants,

C. C. P. Hull, J.P., C.C., Chairman;

C. Holman, M.D.,

Member of Board of Management;

H. Howard, Secretary.

36 King William Street, E.C.

June 1904.

